# Identification of Major Bleeding Events in Postoperative Patients With Malignant Tumors in Chinese Electronic Medical Records: Algorithm Development and Validation

**DOI:** 10.2196/66189

**Published:** 2025-05-01

**Authors:** Hui Li, Haiyang Yao, Yuxiang Gao, Hang Luo, Changbin Cai, Zhou Zhou, Muhan Yuan, Wei Jiang

**Affiliations:** 1 Department of Thoracic Surgery, Beijing Chao-Yang Hospital, Capital Medical University, No.8 South Road of Workers' Stadium, Chaoyang District, Beijing, 100020, China, 86 13701158350; 2 Department of Technology, Shanghai Palan DataRx Co., Ltd, Shanghai, China; 3 Sanofi China Medical Affairs, Sanofi China, Shanghai, China; 4 Pharmaceutical Business Division, Basebit (Shanghai) Information Technology Co., Ltd, Shanghai, China

**Keywords:** machine learning, electronic medical record, postoperative patients with malignant tumors, postoperative bleeding, tumor surgery, abdominal

## Abstract

**Background:**

Postoperative bleeding is a serious complication following abdominal tumor surgery, but it is often not clearly diagnosed and documented in clinical practice in China. Previous studies have relied on manual interpretation of medical records to determine the presence of postoperative bleeding in patients, which is time-consuming and laborious. More critically, this manual approach severely hinders the efficient analysis of large volumes of medical data, impeding in-depth research into the incidence patterns and risk factors of postoperative bleeding. It remains unclear whether machine learning can play a role in processing large volumes of medical text to identify postoperative bleeding effectively.

**Objective:**

This study aimed to develop a machine learning model tool for identifying postoperative patients with major bleeding based on the electronic medical record system.

**Methods:**

This study used data from the available information in the National Health and Medical Big Data (Eastern) Center in Jiangsu Province of China. We randomly selected the medical records of 2,000 patients who underwent in-hospital tumor resection surgery between January 2018 and December 2021 from the database. Physicians manually classified each note as present or absent for a major bleeding event during the postoperative hospital stay. Feature engineering involved bleeding expressions, high-frequency related expressions, and quantitative logical judgment, resulting in 270 features. Logistic regression (LR), K-nearest neighbor (KNN), and convolutional neural network (CNN) models were developed and trained using the 1600-note training set. The main outcomes were accuracy, sensitivity, specificity, positive predictive value (PPV), and negative predictive value (NPV) for each model.

**Results:**

Major bleeding was present in 4.31% (69/1600) of the training set and 4.75% (19/400) of the test set. In the test set, the LR method achieved an accuracy of 0.8275, a sensitivity of 0.8947, a specificity of 0.8241, a PPV of 0.2024, an NPV of 0.9937, and an *F*_1_-score of 0.3301. The CNN method demonstrated an accuracy of 0.8900, sensitivity of 0.8421, specificity of 0.8924, PPV of 0.2807, NPV of 0.9913, and an *F*_1_-score of 0.4211. While the KNN method showed a high specificity of 0.9948 and an accuracy of 0.9575 in the test set, its sensitivity was notably low at 0.2105. The C-statistic for the LR method was 0.9018 and for the CNN method was 0.8830.

**Conclusions:**

Both the LR and CNN methods demonstrate good performance in identifying major bleeding in patients with postoperative malignant tumors from electronic medical records, exhibiting high sensitivity and specificity. Given the higher sensitivity of the LR method (89.47%) and the higher specificity of the CNN method (89.24%) in the test set, both models hold promise for practical application, depending on specific clinical priorities.

## Introduction

Bleeding events are frequent complications encountered in postoperative clinical settings and can stem from the use of anticoagulant or antiplatelet drugs, invasive surgical treatment, or patient-related conditions and the existence of comorbidities, which are associated with increased morbidity, mortality, and health care costs [1-4]. Patients experiencing gastrointestinal bleeding while in the intensive care unit face a fourfold increase in mortality risk compared to those without bleeding issues, along with an additional eight-day stay within the unit [4]. Ample clinical evidence supports the correlation between intraoperative blood loss, especially excessive blood loss and adverse effects on the tumor prognosis. On the other hand, concern about postoperative bleeding may become the main reason why clinicians might be overly cautious in using medications to prevent venous thrombosis (VTE), despite VTE being the second leading cause of death among patients with tumors. While the mortality rate of patients with tumors and VTE is three times higher than that of other patients, timely detection of bleeding risks through progress notes and balanced selection of anticoagulants are crucial for postoperative patients with malignant tumors [5,6].

Currently, there is a growing number of retrospective studies on postoperative bleeding risk in cancer patients. It is also worth noting the lack of fully established risk prediction schemes or risk assessment tools for postoperative bleeding. However, a major challenge in conducting these studies is determining whether patients experienced bleeding events during their past treatment processes. In the clinical setting in China, current approaches to identifying bleeding episodes predominantly rely on diagnostic records, which often lack precision due to inconsistent descriptions and missing details. The task of pinpointing bleeding events from a patient’s medical history can be particularly challenging, especially when dealing with extensive records [7,8]. Despite the transition to electronic medical records, these valuable sources of data are frequently underused. Within these records, details within course notes, including physical examination reports and discharge summaries, often contain firsthand accounts of bleeding incidents or clear indications of their absence. Nonetheless, manual identification of these events can be both time-consuming and arduous, raising concerns about accuracy and practicality. Thus, emphasis should be placed on the imperative need to develop methodologies geared toward effectively identifying bleeding events within existing medical records.

In the realm of clinical research, the accurate identification of bleeding events within large clinical datasets holds paramount importance. Regrettably, the current landscape lacks automated and scalable machine learning (ML) methodologies tailored for this objective. This is a significant unmet need, especially considering the clinical need for early detection of postoperative bleeding to improve patient outcomes. ML has been regarded as a method for developing models that depict intricate nonlinear systems and handling a vast array of potential variables found in contemporary electronic medical records. ML techniques have found application in various health care scenarios such as forecasting cancer susceptibility, automatically categorizing clinical images, and predicting post-transplant prognosis. This is enabled by the abundance of real-world longitudinal datasets derived from the extensive integration of electronic health record (EHR) [9-11]. For health care challenges involving large and complex datasets, especially those with unstructured data, ML methods have demonstrated advantages over traditional statistical regression methods [12,13]. At present, mainstream text recognition methods such as support vector machine (SVM) or random forest (RF) are mostly suitable for large sample size data with high positivity rates, and the recognition of bleeding events may be a process of searching for sporadic positive events in large sample sizes [14,15]. The aim of this study was to develop a machine learning model tool for identifying postoperative patients with major bleeding events based on the electronic medical record system.

## Methods

### Population

This study used a retrospective design. The data for this study were obtained from the National Health and Medical Big Data (Eastern) Center in Jiangsu Province, which is maintained by the Jiangsu Provincial Health Commission. This database includes clinical data from hospitals in Jiangsu province, with sensitive and identifiable information removed to protect privacy. Retrospective electronic medical records data, including clinical notes were used for identifying the study population. We specifically focused on patients with malignant tumor, who underwent surgical procedures between January 2018 and December 2021, reflecting actual clinical scenarios. To more accurately identify high-risk individuals for bleeding, we chose to focus on patients with chest, abdominal and gynecological malignant tumor instead of urinary system tumors. This decision was based on the fact that surgeries for chest, abdominal, and gynecological tumors involve greater surgical trauma, making postoperative bleeding more likely. Additionally, secondary tumors were excluded because they were typically palliatively resected, resulting in a lower risk of bleeding compared to primary tumors.

The primary objective was to develop a classifier capable of recognizing significant cases of bleeding with clinical relevance. To achieve this, we randomly allocated 1600 notes for training purposes and 400 notes for testing the model.

The inclusion criteria in the study population are as follows: (1) patients were diagnosed with primary chest, abdominal, or gynecological malignant tumors; (2) patients underwent surgical resection of the malignant tumor; and (3) patients were ≥18 years old. The exclusion criteria are as follows: (1) patients were diagnosed with urinary system tumors; (2) patients were diagnosed with a secondary tumor; (3) patients only underwent endoscopic surgery (ie, gastroscopy, enteroscopy, cystoscopy); (4) patients diagnosed with bleeding before surgery or who underwent surgery due to bleeding events; and (5) hospitalization course record for the given visit is missing.

Each note was classified as major bleeding present, indicating that clinically relevant bleeding was referenced in the note, or major bleeding absent, indicating that clinically relevant bleeding was not referenced in the note. Major bleeding was defined as fatal or symptomatic bleeding in a critical area or organ or bleeding causing a fall in hemoglobin level of ≥2 g/dL or transfusion of ≥2 units of erythrocyte concentrate, according to the definition of the International Society of Thrombosis and Haemostasis [16].

We included medical history and physical examination notes, progress notes, and discharge summaries that were signed by an attending physician. All investigators with direct access to the data completed a data use agreement. An independent medical professional was selected to read the medical and surgical records and label whether there was bleeding, and the bleeding classification (ie, major bleeding or nonmajor bleeding). Then, another independent medical professional was designated to review the labeled cases and extract or highlight the text content in the medical records where bleeding events occurred for reference in developing bleeding-related regular expressions. Any disagreements or unclear content in the text reading between the two medical professionals were recorded and discussed in an external expert workshop. In the workshop, questionable clinical scenarios were confirmed by two experienced medical experts.

### Feature Engineering

Given the low positivity rate and limited sample size of major bleeding events in the existing data, the methodology for identifying major bleeding events during manual annotation was carefully considered when crafting features. A significant portion of these features was extracted through the application of natural language processing (NLP) techniques like Jieba (for Chinese word splitting) and regular expressions (for text and value extraction). These features are categorized as follows:

The first category: These features were generated from the content of manually annotated major bleeding events in the training set. This set includes 20 key features strongly linked to postoperative bleeding, such as terms like “postoperative,” “ hematochezia,” “ hemorrhagic fluid,” and “treatment with hemostasis surgery.” Furthermore, 241 additional features were compiled by segmenting factors associated with major bleeding events.

The second category: These features were derived by tokenizing the patient’s course texts from the training set using Jieba and arranging them based on their frequency of occurrence. These features represent words that exhibit some relevance to major bleeding events but were not present in the first part. Features in this category were chosen based on a minimum frequency threshold of 900, aiming to complement the initial features and mitigate any shortcomings due to manually crafted features.

**Figure 1.**
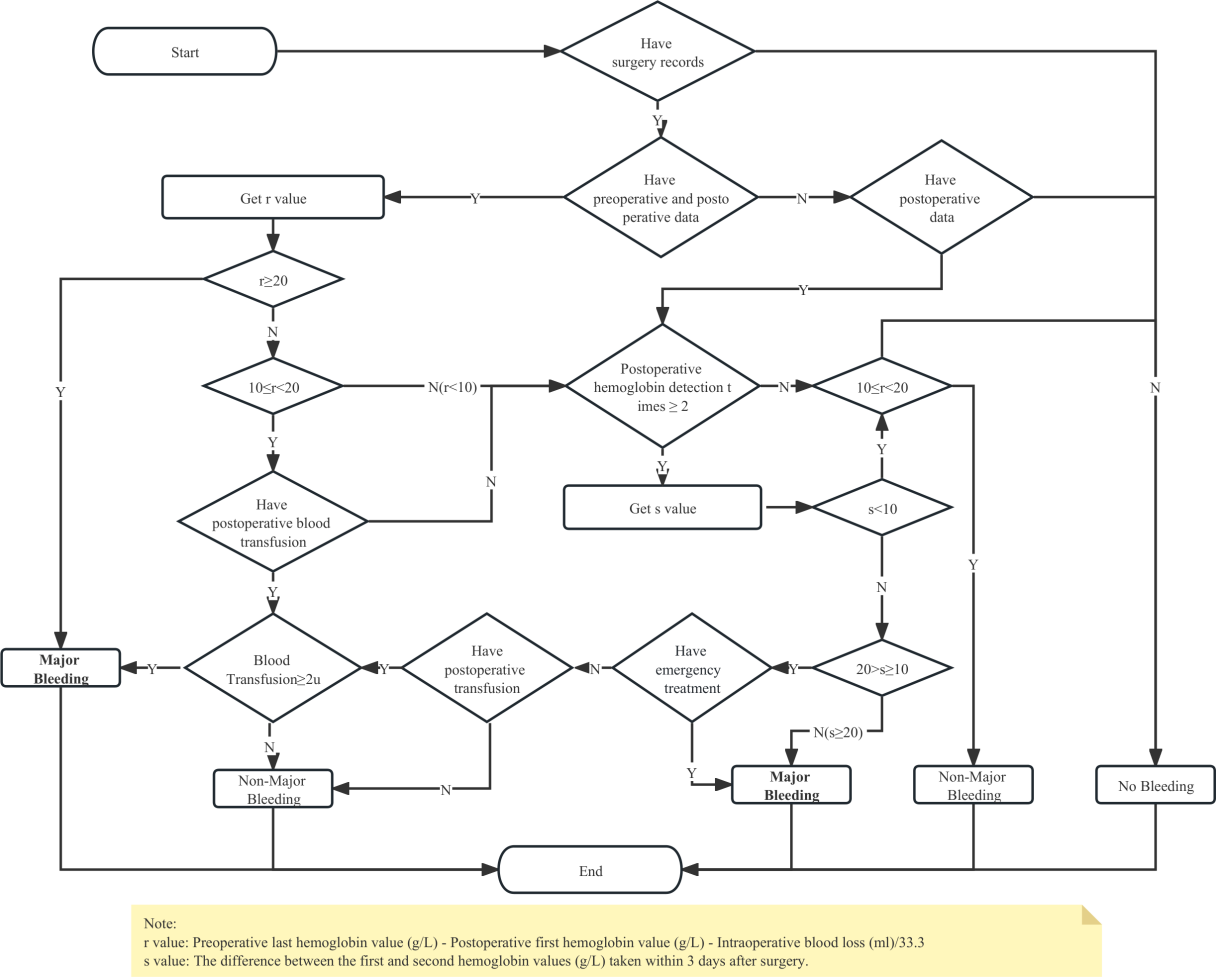
Structured process identification diagram for feature selection.

The third category: This feature reflects insights from clinical experts and primarily revolves around indicators like preoperative, intraoperative, and postoperative hemoglobin levels, bleeding volume, and transfusion volume. They are instrumental in establishing the logic for identifying postoperative major bleeding for each patient. Features in this segment heavily rely on quantitative logical assessments to fill in any gaps present in the preceding two feature groups. For instance, transfusion volumes extracted from structured surgical records and transfusion documentation are analyzed in conjunction with the standardized decision-making flowchart to determine major bleeding status. A detailed, structured process identification diagram is presented in Figure 1.

### Model Training

A supervised learning approach was adopted, where a dataset comprising 2000 patients was divided into a training set of 1600 patients and a test set of 400 patients. Using the features generated through feature engineering and the corresponding labels assigned during manual review, three distinct ML models were successfully trained using different algorithms: logistic regression (LR), k-nearest neighbors (KNN), and convolutional neural networks (CNN).

Here is a breakdown of how each model was implemented:

1. LR:

- Model implementation: developed using Keras
- Architectural details: constructed with a single dense layer
- Activation function: sigmoid function
- Loss function: SigmoidFocalCrossEntropy
- Optimizer: Adam optimizer
- Validation split: 20% of the training dataset is set aside for validation

1. KNN:

- Model implementation: Used the KNeighborsClassifier class from Scikit-Learn.

1. CNN:

- Model implementation: implemented using Keras
- Architectural details: featured 3 hidden layers - a 1-dimensional convolutional layer, a max pooling layer, and a dense layer
- Activation functions: Rectified Linear Unit for the convolutional layer and sigmoid for the output layer
- Output layer: a single dense unit
- Validation split: Again, 20% of the training data is reserved for validation.

All three ML models were trained on the training set consisting of 1600 cases to learn the underlying patterns between the extracted features and the corresponding labels. The use of LR, KNN, and CNN allows for a diverse exploration of the dataset with different algorithms to harness their unique strengths in handling and learning from the data.

### Model Evaluation

Following the training phase, the evaluation of the three ML models—LR, KNN, and CNN—was conducted on the test set containing 400 cases. Various performance metrics were calculated for each model to assess their effectiveness in making predictions. These metrics included accuracy, sensitivity, positive predictive value (PPV), *F*_1_-score, negative predictive value (NPV), and specificity of each model.

After calculating these metrics, the ML models with the highest sensitivity and specificity were chosen for further consideration due to their critical role in the diagnostic accuracy required by the specific use case. By prioritizing both sensitivity and specificity, you ensured a balanced assessment of the models’ performance regarding true positive and true negative rates.

In addition to the confusion matrices, which provide a detailed overview of true positives, false negatives, false positives, and true negatives at the default prediction threshold, we used the receiver operating characteristic curve and the corresponding area under the curve for each model. This comprehensive analysis of the receiver operating characteristic curves and area under the curve values allowed for a deeper understanding of the trade-offs between true positive and false positive rates across different thresholds and enabled the selection of the best-performing models based on a more nuanced evaluation beyond conventional accuracy metrics.

### Ethical Considerations

The study protocol was conducted in accordance with the Declaration of Helsinki and approved by the institutional review board at Shanghai Ethics Committee for Clinical Research (Approval number: SECCR/2023-119-01). The data used in this study were deidentified. Informed consent was waived by the Ethics Committee owing to the use of deidentified data.

## Results

### Baseline Characteristics

The training set represented 1600 patients, of whom 48.5% (776) were female, the mean age was 62.86 (SD 9.16) years, with a total of 33654 course records. The test set represented 400 patients, of whom 49.3% (n=197) were female; the mean age was 62.81 (SD 9.21) years, with a total of 8491 course records. The ratio of postoperative major bleeding was 4.31% (n=69) in the training set and 4.75% (n=19) in the test set (Table 1).

**Table 1.** Population characteristics of training and test sets.

Characteristics	Training set (n=1600)	Test set (n=400)
Gender (female) n (%）	776 48.5	197 49.3
Age (years), mean (SD)	62.86 (9.16)	62.81 (9.21)
Course records, n	33,654	8491
Proportion of major bleeding, n (%）	69 (4.31)	19 (4.75)

### Note-Based Feature Selection

A total of 270 features were selected; first, 261 features related to postoperative bleeding were selected by regular manual disassembly. Second, Jieba was used to segment the disease course text and select 8 features according to word frequency classification. Furthermore, the last feature was created by a logical recognition graph generated from expert opinion. More details are provided in the feature engineering section. The frequency of the top 20 features is shown in Table 2.

**Table 2.** The frequency of the top 20 features selection.

Features (description or regularization in English)	Frequency
Postoperative	1592
(chest | abdomen | pelvis) cavity | rectum. {0,2} depression | posterior fornix | anastomosis | pancreas | digestive tract | stomach | vagina	1579
(out | lose) blood (stop | break into)?| hematoma | subcutaneous Ecchymosis | congestion	1033
Bleeding	1002
Bloody fluid | red.{0,2} drainage fluid	995
Hemostasis	528
Laparotomy	513
Dark red	347
Emergency	343
(To | Perform).{0,15} to stop bleeding	303
Swelling	273
(To | Perform).{0,5} to stop bleeding	253
(((chest | abdomen | pelvis) cavity | rectum.{0,2} depression | posterior fornix | anastomosis | pancreas | digestive tract | stomach).{0,10}bleeding | vagina.{0,2}large .{0,2}(out | lose)blood)	251
Blood loss	246
Pelvic cavity.{0,5}Drainage.{0,5}(bloody liquid | fluid dark red)	225
(?: inject | give).{0,20}(?:suspended less white (?:red blood cell | erythrocyte blood transfusion volume | red blood cell)|whole blood | red blood cell | less white suspended red blood cell | red suspension | suspended less white Red).{1,20}(?:U | ML | CC | ml | u | ml | mL | cc | infusion | unit) | (?: in | out | to).{0,5}(? :U |ML |CC | milliliter | u | ml | mL | cc | infusion | unit).{0,5}(?: suspended less white(?:cell red blood cell | red blood cell transfusion amount | red blood cell) | whole blood | Red blood cells | less white suspension red blood cells | red suspension | suspension less white red)	220
((out | loss) blood | introduction | bloody Effusion).{0,15}(U | ML | CC | ml | u | ml | mL | cc | unit)	220
Lose.{0,20}(red blood cells | whole blood | red suspension)	220
Structured recognition results	206
(exist | has).{0,5} bleed	178

### Comparison and Verification of the Efficiency of Three Machine Learning Models

**Figure 2.**
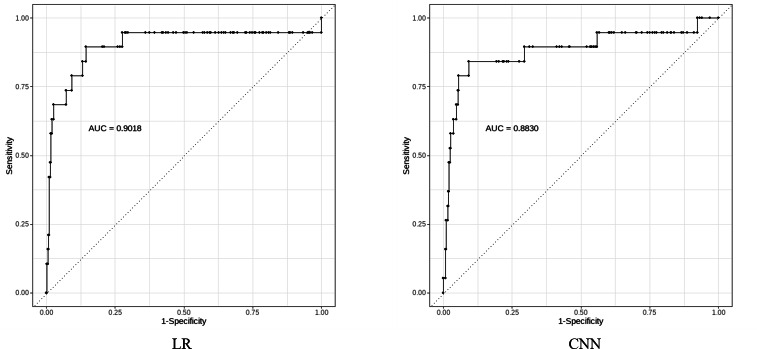
Receiver operating characteristic curves for identifying major bleeding from clinical notes using the LR and CNN methods. AUC: area under the curve; LR: logistic regression; CNN: convolution neural network.

In the identification of major bleeding events within the test set, the LR method had an accuracy of 0.8275, sensitivity of 0.8947, specificity of 0.8241, PPV of 0.2024, NPV of 0.9937, and *F*_1_-score of 0.3301. The accuracy of the CNN method in the testing set was 0.8900, the sensitivity was 0.8421, the specificity was 0.8924, the PPV was 0.2807, the NPV was 0.9913, and the *F*_1_-score was 0.4211. The KNN method had an accuracy of 0.9575, sensitivity of 0.2105, specificity of 0.9948, PPV of 0.6667, NPV of 0.9619, and *F*_1_-score of 0.3200 (Table 3). The C-statistic was higher in the LR method (C=0.9018), followed by the CNN method (C=0.8830) (Figure 2).

**Table 3.** The performance of each model in identifying major bleeding events.

Sets and models	Accuracy	Sensitivity	Positive predictive value	*F*_1_-score	Negative predictive value	Specificity
Training set (n=1600)						
LR^a^	0.8231	1.0000	0.1960	0.3278	1.000	0.8152
CNN^b^	0.9056	0.9710	0.3102	0.4702	0.9986	0.9027
KNN^c^	0.9643	0.1884	0.9286	0.3132	0.9647	0.9993
Test set (n=400)						
LR	0.8275	0.8947	0.2024	0.3301	0.9937	0.8241
CNN	0.8900	0.8421	0.2807	0.4211	0.9913	0.8924
KNN	0.9575	0.2105	0.6667	0.3200	0.9619	0.9948

a LR: logistic regression.

b CNN: convolutional neural network.

c KNN: K-nearest neighbor.

## Discussion

### Principal Findings

In this study, we implemented LR, CNN, and KNN algorithms for ML detection of bleeding events within electronic medical record systems. Notably, CNN was specifically applied to categorize radiology free-text reports and exhibited commendable accuracy [17]. The CNN approach demonstrated a sensitivity of 84% and a specificity of 89% in detecting recorded bleeding events in patients at a threshold of 0.50, while the LR method showcased a sensitivity of 89% and a specificity of 82% at the same threshold. The KNN algorithm can be negatively impacted by high dimensionality in feature matrices. These findings underscore the viability of employing algorithms to pinpoint patients with bleeding events within extensive textual disease records.

### Evaluation Metrics and Application Context

*F*_1_-scores are frequently used in the evaluation of classifiers. The *F*_1_-score can be considered the harmonic mean between the precision and recall (sensitivity) [18]. Thus, it symmetrically represents both precision (how accurately the model identifies only true positive instances) and sensitivity (how well the classifier identifies all actual positive instances) in one metric. Although the three machine learning models in this investigation did not attain high *F*_1_-scores on the test set, it must be clarified that the goal of the investigation was to detect bleeding events as accurately as possible in practical applications for subsequent manual assessment. Therefore, greater attention is paid to the sensitivity and specificity (ie, how well the classifier identifies all actual negative instances) of the model, the high values of which imply that the model can detect hemorrhagic events while avoiding false alarms, which is crucial for this investigation. Consequently, priority is placed on the models’ sensitivity and the attainment of greater specificity. Hence, we maintain that the models developed in this study remain valuable for identifying bleeding events within disease records and represent a promising avenue for future research endeavors.

### Comparison to Prior Work

The integration of NLP and ML has proven successful across various domains. For instance, in the context of health care, predictive models leveraging ML and statistical methods have demonstrated the ability to forecast occurrences such as postpartum hemorrhage upon labor admission with reasonable discriminatory power. A prior study demonstrated the effectiveness of a Hybrid CNN-LSTM Autoencoder model for the detection of bleeding events within EHR data. This was accomplished through the integration of a supervised CNN with a pretrained, unsupervised Bidirectional Long Short-Term Memory autoencoder. The primary objective was to accurately predict the presence of a bleeding event within a given English sentence from an EHR record [19].

While prior research primarily focused on English EHR data and general bleeding event detection, such as the study by Li et al that used a Hybrid CNN-LSTM Autoencoder for sentence-level bleeding detection, and a more recent work that applied retrieval augmented generation with large language models for detecting nonsurgical major bleeding events in English EHRs [20], our investigation centers on the identification of major bleeding events within Chinese EHR. Chinese NLP presents inherent complexities, especially in word segmentation within EHR text, posing a significant hurdle. Furthermore, the shift in focus to major bleeding, which exhibits a considerably lower incidence rate compared to general bleeding events, substantially reduces the availability of positive samples, thereby intensifying the challenge for robust predictive modeling. Building upon these complexities, unlike the prior studies’ sentence-level analysis, our research takes a holistic approach to identifying major bleeding events within the entire patient visit context. This broader, patient-centric perspective further complicates the analysis and requires a more comprehensive understanding of the clinical narrative. These critical distinctions—language specificity, event granularity, and analytical scope—collectively highlight the greater challenges of our study compared to existing work in the field.

### Strengths and Limitations

A key strength of this study lies in the strategic approach to model selection and feature engineering. Initially, SVM and RF algorithms were explored. However, recognizing their reliance on effective word segmentation and the challenges posed by the heterogeneous nature of the text data, the focus was deliberately shifted to LR, KNN, and CNN. To further enhance model performance, term frequency-inverse document frequency (TF-IDF) scoring was effectively used to compute feature representations, addressing the complexities of free-text data. Moreover, to overcome the limitation of a relatively small sample size, particularly the scarcity of positive samples (fewer than 100), and the resulting dispersed TF-IDF features, a targeted feature engineering approach was strategically adopted. This involved selecting predefined features associated with bleeding based on expert medical domain knowledge. This approach allowed the ML models to concentrate on clinically relevant features, represented as binary values (0 and 1), rather than solely relying on traditional TF-IDF metrics applied to the entire text. This targeted feature engineering proved crucial for enhancing performance in a limited data setting. As a result of these strengths, including strategic model selection, targeted feature engineering, manual annotation, and training with limited samples, the LR and CNN models demonstrated superior performance compared to SVM and RF models based on TF-IDF features for detecting major bleeding events. This aligns with findings from similar research, as evidenced by a study that underscored the enhanced performance of CNNs when expert annotation of text data is incorporated [19], further validating the efficacy of CNNs in text classification tasks within the medical domain.

However, manual feature selection has certain limitations. First, engineers must dedicate time to writing code for feature extraction based on the definitions provided by the medical field, which is a more time-intensive process compared to the automatic feature computation performed by machines. To partially mitigate this, we prioritized high-impact features identified through clinical expert consultations, but future studies could integrate semiautomated pipelines to balance efficiency and domain specificity. Second, as previously discussed in related research [21], the identification of postoperative bleeding and feature selection depends on manual procedures, thereby increasing the likelihood of bias and oversight. While dual annotations by multiple clinicians were used to reduce subjectivity, discrepancies were resolved through consensus rather than quantitative metrics, potentially affecting reproducibility. Future work should adopt standardized annotation protocols with inter-rater reliability assessments. Third, the chosen features encapsulate domain-specific knowledge, sometimes being closely tied to specific hospital departments with varying requirements and documentation practices in electronic medical records. Broader applicability requires multicenter collaboration to harmonize feature definitions across institutions. Moreover, our study included a retrospective analysis with a relatively limited number of specimens, a factor to be considered for broader applicability in ML analyses. Similar challenges have been observed in other comparable studies [19]. Consequently, not only does this constrained universality impede application in diverse fields, but also when extending to other illnesses, hospitals, and departments, there is often a need to reimagine and recreate the features.

### Conclusions

Based on our new text feature selection method, both the LR and CNN methods perform well in identifying major bleeding occurring in postoperative patients with malignant tumors from electronic medical records.

## Abbreviation

CNN: Convolutional neural network
KNN: K-nearest neighbor
LR: Logistic regression
ML: Machine learning
NLP: natural language processing
NPV: negative predictive value
PPV: positive predictive value
RF: random forest
SVM: support vector machine
VTE: venous thrombosis
